# Analectic

**Published:** 1834

**Authors:** 


### MISCELLANEOUS INTELLIGENCE.

### ANALECTIC, ANALYTICAL, AND ORIGINAL.

### I. Analectic.

*Ad Sylvas Nuncius.*

1. *Sympathy between the Uterus and Mammæ.*—The *London Medical Gazette*, for March last, contains some observations by Dr. Rigby, on this subject, which are peculiarly interesting, from the practical advantage to be derived from the play of this sympathy in the suppression of uterine hæmorrhage. There is, perhaps, remarks Dr. R. "nothing more interesting, or more worthy the attention of an accoucheur, than the various sympathies which show themselves between the uterus and other parts of the system. The morning sickness of early pregnancy; the convulsions in the latter months, or during labor; the violent rigors when the os uteri becomes fully dilated, or immediately after the birth of the child; the contraction of the uterus when the face is sprinkled with cold water, or after a draught of any cold fluid; are some of the most remarkable, among a considerable number, which occur before and during labor.

"The connexion, in the unimpregnated state, between the uterus and stomach, is well displayed by the gastric derangement which frequently accompanies prolapsus uteri, and *vice versâ*. The pain and swelling of the mammary gland, from menstrual irritation, or where there is subacute or chronic inflammation, or organic disease of the uterus, in like manner demonstrate the link between this organ and the breast. But the most striking instance of sympathy which I have remarked between these two organs, is the sudden and powerful contraction which is excited in the uterus, when in a state of inertia, by applying the child to the breast.

"My attention was first drawn to this subject, by an observation which I met with some years ago, in Carus's *Gynakologie*, recommending the application of the child to the breast, to promote expulsion of the placenta, where it was slow in coming away; but I was far from being aware of the extent to which this sympathy really existed. In cases where there has been considerable disposition to hæmorrhage, after labor, from noncontracted uterus, and where I have been afraid to leave the patient, lest flooding should come on in my absence, I have been for the last two years, in the habit of ordering the child to be put to the breast, as soon as her clothes, &c. were changed, and herself comfortably settled in bed; feeling that I thus diminished the chances there might be of any hæmorrhage occurring. It was not however till last year, that I began to see the practical importance of this sympathetic connexion, between the breast and uterus, in its full extent. Having had two or three cases of severe hæmorrhage after labor, from uterine inertia, which had to a degree resisted all the common modes of treatment, and where permanent contraction could not be induced, even by repeated injections of cold water and vinegar into the vagina; I determined to see what effect the application of the child to the breast would have upon the relaxed uterus, and was agreeably surprised to find the observation of Professor Carus confirmed in its fullest extent; firm and permanent contraction having been immediately produced, in every case."

"When I first began," Dr. R. adds, "to use this plan of treatment, in cases of inertia uteri after delivery, I was frequently startled by finding a sudden gush of blood, accompanied by a forcible discharge of coagula, follow almost instantly the application of the child; but soon satisfied myself, that so far from being a return of the flooding, it was merely the result of the uterus contracting firmly and expelling its contents.

"It is a common saying among nurses, that 'the child brings after pains;' that is, when the child is first applied to the breast, on the second or third day, as was formerly the custom, it was frequently followed by pretty smart after pains, which evidently resulted from the uterus being excited to contract, and expel any coagula of blood, which might be lodging in its cavity or sinuses. Hence, besides its beneficial effects, in preventing any danger from hæmorrhage, the application of the child to the breast, is a valuable means of preventing much of that uterine pain and irritation, which is apt to result from the presence of coagula, &c. in the womb, after labor.—*Am. Jour. Med. Sciences.*

2. ***Memoir upon the Structure of the Lymphatic Vessels. Read at the Medical Society of Emulation, of Paris, at its meeting on the 2d of October***, 1833. By Professor Mojon.—Dr. M. former Professor of Anatomy and Physiology, at the Royal University of Genoa, has lately made some new observations upon the structure of the lymphatic vessels, which appear to be well worthy of the interest of physiologists. Having placed the lymphatic vessels upon a glass plate, and opened them through their entire length, Dr. M. has recognised, by the aid of the microscope, that which anatomists regard as valves or folds of their internal membrane, are in fact true sphincters. These sphincters are formed by circular fibres, which, diminishing at different points the calibre of the lymphatic tube, give rise to those nudosities which are remarked at their exterior. The contractions are still more visible when the lymphatics are injected with some liquid. They may also be observed very distinctly when this system is in an almost varicose state, as in subjects who have died with anasarca.

If the two ends of a varicose lymphatic, be drawn in a contrary direction, these external nudosities disappear entirely, as well as the pretended internal valves.

Professor Mojon has observed, besides, that the fibrous membrane of the lymphatics, of which Mascagni has spoken with sufficient exactness, has longitudinal filaments from one contraction to another, much more numerous than the oblique. This crossing of the fibres forms a tissue, like a kind of lattice.

The longitudinal fibres have their two ends attached to the transverse, which constitute, according to the views of the Genoese anatomist, the sphincters or contractors of the lymphatics. Thus the longitudinal fibres in contracting, draw one sphincter nearer to another, whilst the oblique fibres diminish the diameter. All these fibres, taking their point of support upon the circular fibres, dilate the superior sphincters, by drawing the circumference downwards. By means of this physico vital mechanism, the fluid which penetrates a lymphatic, irritates the portion of the vessel which it fills, which contracting upon itself, diminishes its cavity, and the fluid there contained is obliged to advance by thus successively traversing the open sphincter. This peristaltic motion is performed in the same manner as that of the intestines. This vermicular movement may be observed very distinctly in the lacteal vessels of the mesentery of animals, which are opened two or three hours after they have been well fed.

By admitting this organization of the lymphatics, the retrograde movement of the fluids contained in the absorbent system, admitted by Darwin and others, may be explained, which would be incompatible with a valvular apparatus.

If this system of vessels were valvular, why, says Dr. Mojon, when a lymphatic is opened in its whole length, does it never present but two parallel crescents, from one space to another, one to the right, the other to the left, and never one and two halves? That, indeed, would happen often, if these crescents were true valves, like those of the veins.

The difficulty which is often met with in attempting to inject the lymphatic vessels in a contrary direction to the fluid which passes through them, is owing to this; that the little pouches formed by the sphincters, and the relaxation of their parietes in filling them with the injecting matter, inflate them, and by that means close the opening of the lymphatic.

The observation several times repeated, that the different colored fluids with which the lymphatic vessels are injected, never spread themselves either in the cellular tissue, or in the parenchyma of the viscera, unless by some laceration, induced M. Mojon to believe that these vessels had no patulous orifice, and that they took their origin from a cellular filament, which progressively became a villosity, an areolar spongiole, a capillary, and at length a lymphatic trunk. He also believes, that the absorbent action of this system of vessels, is performed by a kind of imbibition, through the porosity of their most delicate branches, like a sponge.

When once the liquid has penetrated by this kind of endosmose into the cavity of the smallest branches of the lymphatics, it advances into the larger trunks, by a progressive and continued peristaltic motion, up to the absorbent system.

Several French anatomists have lately repeated these experiments; they have obtained the same results.—*Journal de la Societe des Sciences Physiques et Chemiques, Nov.* 1833.—*Am. J. Med. Sciences.*

3. *On the Pathology of Typhus Fever.* By Professor Bouillaud.—It results, says M. Bouillaud, from the thirty-six cases of typhoid entero-mesenteritis, which were lately treated in the wards of the La Charité, that the inflammatory affection of the lower part of the small intestines, and especially of the clusters of the glandulæ Peyeri, constitutes really and truly the fundamental and essential element of the disease. In every instance, from the very commencement of the morbid phenomena, the local symptoms have clearly indicated the existence of such a phlegmasia; and the typhoid state has been developed under the influence of the entero-mesenteritis, in the same manner as we see it supervene in certain cases of severe phlegmonous erysipelas, of phlebitis, &c. It would not be more reasonable to consider inflammation of the intestinal follicles and of the mesenteric glands, as a simple consecutive effect of the typhoid state, than it would be to regard a phlegmonous erysipelas, in the course of which typhoid symptoms were developed, as the result of these very symptoms. Such a doctrine would be, in truth, a "contre-sens," pathogenesis. We do not indeed deny, that erysipelas may occur in subjects already laboring under typhus fever; all that we contend for is, that the obverse case, viz. where the erysipelas is formed before the explosion of the typhoid symptoms, is by no means uncommon; and the scope of our reasoning is obvious, when we assert, that the entero-mesenteric phlegmasia is of an erysipelatous character. To those who gainsay our doctrines, we confidently challenge them to adduce a single well recorded and well authenticated case of acute inflammation of Peyer's, and of the mesenteric glands, which did not exhibit, in its march and in its symptoms, local as well as constitutional, a most close analogy, if not a complete identity, with that disease, which has been most unfortunately designated by the appellations of fever, typhoid affection, &c. If it be true, as the Father of Medicine has predicated, that "naturam morborum ostendit curatio," another very potent argument may be adduced in favor of our pathological tenets, from the success of the remedial measures we adopt. These are eminently antiphlogistic. It may be said that some of the means, as the quinine and the chlorurets, (which were used in a few of the cases in the hospital,) are very far from being so; but who does not perceive, that at the period when we had recourse to these, the inflammatory affection had assumed what the ancients denominated a malignant, or rather a putrid character, and therefore required for its arrest the intervention of certain measures, superadded to those of a strictly antiphlogistic tendency? In the period of the malady to which we are referring at present, there is indubitably a focus of putrid decomposition, which reacting on the whole economy, induces great and important changes in the mass of the blood and other fluids, and to counteract the effects of which, a new and paramount indication arises—an indication which is best fulfilled by the use of the chlorurets, both externally and internally, and of certain tonics, especially quinine.

How agreeable it is to find out that the doctrines which we have taught and inculcated for so many years, are in accordance with the experience of some of the greatest men who have gone before us. The illustrious Sydenham, whose authority is so often misapplied and abused in the present day, has admirably pointed out the relations between the phenomena of malignancy or putridity, and certain sorts or shades of inflammatory action:—"Cujus de malignitate opinionis inventio, humano generi longe ipsa pyrri-pulveris inventione lætalior fuit. Cum enim hæ febres præsertim malignæ dicantur, in quibus intensioris præ cæteris inflammationis gradus conspicitur." The following case will illustrate my treatment of typhus gravior.

A man was brought to the hospital, in the second week of typhus fever, and of such an aggravated character were all the symptoms, that we quite despaired of saving him. The prostration was extreme—the tongue, lips, and teeth, covered with a black crust—breath very fetid—respiration exceedingly feeble—pulse minute, and very rapid—abdomen distended—slight diarrhœa—surface of the belly and chest exhibited several reddish patches and papulæ, (eruption typhoide.) The state of this patient positively forbad the employment of any depletory measures, and the only judicious indication seemed to be, to obviate the putrid symptoms by the use of the chlorurets in drinks, baths, and enemas. In three or four days there was a sensible amendment; a blister was applied on the calf of each leg, and ten grains of the sulphate of quinine sprinkled every day on the excoriated surfaces. The diet was gradually rendered more nourishing, and consisted of broths, soups, fruits, and weak wine and water. This patient ultimately recovered.

Let the preceding case satisfy my opponents, that the same treatment is not uniformly, and to the same extent, followed, in my treatment of fever, without regard to the character of the symptoms, or to the constitution of the patient. By a judicious combination of antiphlogistic and antiseptic remedies, thirty-three cases, out of thirty-six, admitted into our wards, were saved. Such success could not rationally be expected to result from a therapeutics, which inculcated the use of stimulants, purgatives, and emetics. At the commencement, indeed, of the malady, a purgative or an emetic may be administered with advantage; but if they be of drastic severity, or are frequently repeated, the enteritic evil must necessarily be much aggravated.—

What confirms me in this opinion is, that I know that M. Trousseau has lately abandoned the practice of giving frequent doses of Glauber's salt, in dothinenteritis, [the entero-mesenteritis typhoide,] as recommended by his master, M. Bretonneau. At present he appears to be satisfied with the "medicine expectante;" for he gives nothing else but the white oxyde of antimony, a substance very nearly quite inert; and yet he assures us, that his success of late has been very great in the treatment of cases of genuine typhus. No doubt much good may arise from merely abstaining from every thing positively injurious, and from not interfering with nature's own operations; but we think that even the late M. Dance, who was very sceptical as to the advantages of the common practice in fevers, could not have withstood such practical evidence as we have adduced in favor of the plan which was followed in the thirty-six cases, of which no fewer than thirty-three recovered.—*Jour. Hebdomadaire.—Am. J. Med. Sciences.*

4. M. Bouillaud *on Follicular Enteritis.*—The term "follicular enteritis," is to be preferred to the one formerly in use, "gastro-enteritis," on account of its more accurately indicating the nature of the existing lesion; for in many cases there is no inflammation of the stomach, the disease being limited to the mucous membrane, and especially the follicular apparatus of the small intestines; and besides, we see every day, examples of genuine gastro-enteritis, unaccompanied with the symptoms of typhus fever. M. Bretonneau has lately introduced the term "dothinenteritis," or furuncular enteritis; and, although we cannot with strict propriety, admit the furuncular character of the disease of the intestinal glands, the term is not a bad one. Follicular enteritis has the same meaning, and is more simple in its enunciation. The bizarre appellation of "ileo-dicliditis," in allusion to the seat of the disease in the ileum and ileocœcal valve, does not at all express the nature of the diseased change, but rather its mere habitat. M. Bouillaud is in the habit of employing the terms of adynamic or putrid entero-mesenteritis, and in order to localize the chief seat of the morbid change, adds occasionally a special, instead of the general prefix, thus, adynamic ileo-mesenteritis, &c.

Eighteen cases occurred in the service of M. B. at the La Charité, during the summer months, and of these, fifteen took place in men, and the remaining three in women. This striking difference is, no doubt, attributable to the more debauched and irregular lives of the former, and also to the circumstance of far more men coming to Paris, from the country or elsewhere, for subsistence; for indeed the greater number of the cases we see here, are in those who have resided but a short time in the city. Out of our eighteen patients, eleven had been only four months in Paris—four from six to twelve months—one fifteen and another twenty months resident there. They were all under twenty-eight years of age. The season of the year, viz. during the months of June, July, and August, is that when the follicular enteritis is usually most frequently seen. It is always a difficult, and often an impossible thing, to trace the exciting cause of fever, to any one specific agent or influence; and we shall be generally correct, if we enumerate, not one, but several, such as recent arrival from the country, laborious and excessive work, unwholesome and acrid food, debauchery, exposure to the inclemencies of the weather, depression of spirits, &c.

The premonitory symptoms are, a greater or less prostration, general uneasiness, anxiety, want of appetite, feeling of coldness over the surface, thirst, headache, and in many cases, some degree of purging and nausea, or even vomiting. The headache becomes more and more severe, accompanied with noises in the ears, appearance of flashes of light before the eyes, vertigo, stupor, so that questions are answered slowly and with reluctance, and in many cases with delirium. The features are void of animation, except when inflamed during the delirious paroxysm; the eyes become considerably sunk, the nose is sharpened, and the lips are black, and coated with dark crust. The skin is always hot and dry—the gentlest perspiration is ever a most favorable symptom; for, in all the worst cases, there is a constant aridity of the surface, and only towards the latter stage is it bedewed with an offensive sickening moisture. In some cases, there is an eruption on different parts of the body; this may be either papular, exanthematic, or pustular. A very characteristic symptom of this fever, when severe, is the position or decubitus of the patient in bed—he lies in one attitude, seemingly unconscious of all around him, and if he moves, it is rather like the rolling of a senseless mass than the voluntary act of an animate being. Complete coma, convulsions, twitching of the tendons, &c. are always very unfavorable signs.—The tongue is generally smooth, dry, and red at the point and along the edges, in the early stage; a filthy, yellow colored, cheesy looking crust covers it; and in the more severe cases, so completely parched is it, that it has the appearance of having been broiled. The lips, teeth, and mouth, are invested with a filthy brown or black sordes, and the breath is offensively disgusting. The thirst is always great—sometimes excessive. The gastric irritation is by no means observed in most cases; thus, in our eighteen patients, six only experienced vomitings; and except in one case, there was not any tenderness of the epigastric region when pressed upon. Pressure on the abdomen, especially over the cœcum and ascending colon, produced pain in ten cases. In fourteen there was well marked meteorism, or inflation of the bowels; and the degree of this symptom was usually proportionate to the severity of the case. The involuntary discharge of the urine, especially if this be muddy, ammoniacal, and fetid, always prognosticates great danger. The almost uniform coexistence of pulmonary disease, deserves our serious notice; in fifteen of our cases there was acute bronchitis, indicated by a dry sibilant, or mucous sibilant râle; in two, severe cynanche existed, and in one a double pneumonia, which caused the death of the patient. The biliary apparatus was not much affected in the majority of the cases. Four of the patients died, and the following are the most striking necroscopic appearances found.

Serosity under the membranes of the brain; substance of the brain more highly injected than usual; consistence nearly normal. The stomach, in all cases, presented at one or more points, arborizations of minute vascularity; the texture of the mucous lining was generally softened, and thinner than in health. The duodenum was but little affected. On examining the ileum, there were found, sometimes even from its very commencement, patches of distinct eruption, or of an intense vascularity, with intermediate portions of healthy surface; but these phenomena were always more distinct as we approached its lower or cœcal extremity; at first, or highest up, the glandulæ Peyeri and aggregated follicles were merely swollen and enlarged; then here and there they were found to be ulcerated, the little ulcers having thickened edges, and the subjacent cellular tissue being denuded; as we proceeded lower down, the glandulæ Peyeri became more developed, and around them, the single or isolated follicles were converted into aphtous like ulcerations, with red, and even bloody borders; towards the extremity of the ileum, the gut was found to be actually riddled with these ulcerations, which were surrounded with the soft, pulpy, and inflamed mucous membrane. In one case, the surface of the ileo-cœcal valve, and also of the cœcum, exhibited these last described appearances. The mesenteric glands, adjoining to the diseased portions of intestine, were red, hypertrophied, and softened. Most of the other viscera, as the liver, spleen, heart, &c. presented more or less ramollissement of texture, so that they were easily crushed between the fingers.

As to the *treatment*, it was in all cases of an antiphlogistic tendency —moderate, but not repeated venesection; the application of leeches upon the epigastric and cœcal regions, (the *average* number required for each patient was between sixty and seventy in all, or about sixteen at four different times;) in four cases, cupping was employed to subdue the bronchitic affection. In nine of the cases, blisters were used, sometimes to the thighs, at other times to the calves of the legs. In ten cases, the solutions of the chlorides, (which were first recommended in 1826, by M. Bouillaud, as valuable remedies against the intestinal disease, and the consecutive alteration of the mass of blood,) were employed, either by the mouth, or in injections, or lastly, mixed with poultices, and applied to the abdomen. The bed clothes also, of the patients, were freely sprinkled with them.—*Med. Chirurg. Rev. and Journal Hebdomadaire.—Am. Jour. Med. Sciences.*

5. *On Chronic Gastritis.* [Extracted from Dr. Stokes' Clinical Lectures.]—Chronic gastritis is an extremely interesting disease, whether we look upon it with reference to its importance, its frequency, or its Protean character. It is commonly called dyspepsia, and this term, loose and unlimited in its acceptation, often proves a stumbling block to the student in medicine. Dyspepsia, you know, means difficult digestion, a circumstance which may depend on many causes, but perhaps on none more frequently than upon chronic gastritis. In the great majority of dyspeptic cases, the exciting cause has been over stimulation of the stomach, either from constant excess in strong, highly seasoned meats, or indulging in the use of exciting liquors.—Persons who feed grossly and drink deeply, are generally the subjects of dyspepsia; by constantly stimulating the stomach, they produce an inflammatory condition of that organ. Long continued functional lesion will eventually produce more or less organic disease; and you will find, that in most cases of old dyspepsia there is more or less gastritis. But let us go further, and inquire whether those views are borne out by the ordinary treatment of dyspeptic cases. When you open a book on the practice of physic, and turn to the article dyspepsia, one of the first things which strike you, is the vast number of cures for indigestion. The more incurable a disease is, and the less we know of its treatment, the more numerous is the list of remedies, and the more empirical is its treatment. Now, the circumstance of having a great variety of "*cures*" for a disease, is a strong proof, either that there is no real remedy for it, or that its nature is very little understood. A patient afflicted with dyspepsia will generally run through a variety of treatment; he will be ordered bark by one practitioner, mercury by another, purgatives by a third; in fact, he will be subjected to every form of treatment. Now, all this is proof positive, that the disease is not sufficiently understood.

What does pathology teach in such cases? In almost every instance where patients have died with symptoms of dyspepsia, pathological anatomy proves the stomach to be in a state of demonstrable disease. It appears therefore, that whether we look to the uncertainty and vacillations of treatment, or the results of anatomical examination, the case is still the same; and that where dyspepsia has been of considerable duration, the chance is that there is more or less of organic disease; and that if we prescribe for dyspepsia, neglecting this, we are very likely to do mischief. I do not wish you to believe that every case of dyspepsia is a case of gastritis. This opinion has brought disgrace on the school of Broussais. His disciples went too far, for whether the gastric derangement depended on nervous irritation, or anæmia, or disease of the liver, or mental emotion, they prescribed leeches and water diet, and thus very often brought on the disease they sought to cure. We may have functional disease, independent of structural lesion in the stomach, as well as in any other organ; it is no unusual circumstance, and the practical physician meets with it every day. A great deal of confusion, however, arises from the similarity of the symptoms. I remember an accomplished friend of mine getting into disgrace with one of the members of a board of examiners, on this subject. He was asked to tell the difference between the symptoms of chronic gastritis and dyspepsia, and in reply stated that he could not. For this he was nearly rejected; but I believe, on a candid review of the circumstances, you will agree with me, that he knew more of the matter than the learned professor. In ninety-nine cases out of a hundred, of chronic gastritis, there is no fever, scarcely any thirst, often no fixed local pain; and this leads persons away from an idea of the existence of an inflammatory condition of the stomach.—What are the symptoms of a chronic gastritis? Pain of occasional occurrence, flatulence, acidity, swelling of the stomach, fœtid eructations, sensation of heat and weight about the epigastrium, and perhaps vomiting. Well, these are also the symptoms of dyspepsia, whether it be accompanied by inflammation or not.

How then, when called to a case of this kind, are you to determine the point? I must mention to you here, that it is often hard to do this with certainty. There are two circumstances, however, which you should always bear in mind, as they will afford you considerable assistance in coming to a correct diagnosis; *first, the length of time which the disease has lasted;* secondly, the result of the treatment which has been employed. You will find, that where the disease is a chronic gastritis, that it has been of some duration, that it has come on in an insidious manner, and that it has been exasperated by the ordinary treatment of dyspepsia. Many persons think, that if you give a patient medicine, without regulating his diet or issuing a prohibition against full meals, that you can cure him; and that as he has no fever, and can go about his usual business, there is no necessity for antiphlogistic regimen. But as the disease goes on, he complains of pain in the stomach during the process of digestion, feels uneasy after dinner, there is an unpleasant degree of fullness about the epigastrium; he also experiences a variety of disagreeable symptoms, sometimes being annoyed with pain in the chest, sometimes he says he feels it in the region of the heart, and sometimes about the cartilages of the eighth and ninth ribs. These symptoms subside after the process of digestion is completed, but during its continuance they harass the patient. Very often relief is obtained by vomiting, and hence some persons are in the habit of throwing up their food for the purpose of relieving themselves, and consequently can have no benefit by it.—In some cases digestion goes on until the food seems to reach a particular point, and then an acute feeling of pain is experienced. In these cases the gastritis is generally circumscribed, and is likely to terminate in circumscribed ulceration. Various fluids are rejected from the stomach, during the course of a gastritis; sometimes acid, sometimes alkaline, sometimes insipid and sweet, sometimes bitter and bilious. There is generally a degree of fullness about the stomach, and the epigastrium is tender on pressure, but no decided tumor, either of the pylorus, liver, or spleen, although the epigastrium presented that appearance of fullness and tension, termed by the French "*renitence.*" The bowels too, are constipated, and this is a matter worthy of your attention, for it sometimes unfortunately happens, that the practitioner, mistaking the gastritis for simple constipation, goes on prescribing purgative after purgative, until the patient gets incurable disease of the stomach. I know a case of a lady who gets one stool a week by taking eight drops of croton oil. Some years ago, she was in the enjoyment of excellent health; her bowels happened to get confined, and she was treated by a systematic practitioner with continued purgatives; her bowels are now completely torpid, except when they are subjected to this unnatural stimulus. There are thousands of persons treated in this way, because practitioners look to consequences, and not to causes.

There is one remarkable difference between acute and chronic gastritis, which deserves your attentive consideration, as it exemplifies a law applicable to all viscera under similar circumstances; and this is, that the sympathetic irritations are not so frequent or distinct in chronic inflammation as in the acute form; and hence, in a case of chronic gastritis, we almost never have fever, and the affections of the nervous respiratory or circulating systems, are by no means so well marked. It may even go on to actual disorganization of the stomach, and yet the patient will not complain of any particular symptom during its whole progress, which you could set down as depending exclusively on the sympathetic irritation of gastritis.—Some of these cases, called dyspeptic phthisis, by Dr. W. Philip, are most probably examples of the sympathetic irritation of the lungs from chronic gastritis.

Another case, respecting which much error prevails, is what has been called hypochondriasis. Persons laboring under these affections are condemned to run the gauntlet of every mode of treatment; sometimes (and fortunately for themselves) they are sent to travel, sometimes they are treated with musk and antispasmodics, then with the mineral acids, then with purgatives and mercurials, and lastly with bark, nitrate of silver, and stimulants. They go about like spectres, from one practitioner to another, trying remedy after remedy, alternately sanguine with hope or saddened by disappointment, until at last they die; and, to the astonishment of all the doctors, the only disease found, on dissection, is inflammation and thickening of the mucous surface of the stomach. A condition which, under these circumstances, it was difficult to say whether it was the original disease, or produced by "*fair trials*" of a number of powerful agents. Hypochondriasis is not always gastritis; but it is now found, that in many cases it commences and terminates with disease in the upper portion of the digestive tube and the assisting viscera. This you must always bear in mind.

Chronic gastritis terminates in various ways. Sometimes the inflammation is limited to a particular spot of the stomach, and here we frequently discover circumscribed ulcerations. In very bad cases these ulcers go on perforating the various coats of the stomach, until at last the contents of that organ escape into the serous cavity of the abdomen, and the patient rapidly sinks under a fatal peritonitis. It does not follow, however, that in all cases of perforation, the contents of the stomach get into the perineum, causing death. Very often adhesions are formed, and the base of the ulcer is the serous covering of some other portion of the digestive system, or a false passage may be formed into the colon. One of the most common terminations of a chronic gastritis is, that the inflammation extends to other viscera; the patient gets disease of the liver, spleen, peritoneum, or lungs, and sinks under a complication of disorders. It was somewhat in this way that Napoleon died. He labored for a considerable time under chronic disease of the stomach, which seems to have been overlooked by his medical attendants, and this terminated in the extension of disease to various other organs.—*Lond. Med. and Surg. Jour., Jan. 25th*, 1834.—*Am. Jour. Med. Sciences.*

6. *Treatment of Chronic Gastritis.* By William Stokes, M. D. [Extracted from Lectures on the Practice of Medicine.]—The first thing you should do, when called to treat a case of dyspepsia, is to ascertain whether it be a purely nervous disease, or a chronic gastritis. The majority of practitioners give themselves no trouble about this matter, not recognising the fact, that of the number of dyspeptic persons who seek for medical advice, a considerable proportion are really laboring under a chronic gastritis, and forgetting that in consequence of long continued functional injury, what was at first but a mere nervous derangement, may afterwards become complicated with organic disease. You must also bear in mind, that the stomach is perhaps placed under more unfavorable circumstances for bringing about a cure than any other organ, because the life of the individuals demands that the stomach, though in a state of inflammation, should still continue to perform its functions. In treating diseases of other organs, you will have the advantage of a comparative state of rest; but in a case of the stomach, if you wish to preserve life, you cannot prohibit nutriment, and consequently you must run the risk of keeping up these periodic vascularities which its condition requires, which, though harmless in health, become a source of evil when the stomach is diseased. The obvious deduction from this is, that the cure of a chronic gastritis depends as much upon regimen as upon medical treatment; and particularly where the symptoms have arisen from long continued excitement, as in the case of persons who live highly. Here the treatment chiefly depends on regulating the diet, and if your patient has sense enough to live sparingly for a few weeks or months, you may be able to effect a cure without other treatment. The great error is, that most practitioners attempt to cure the disease by specifics, and when these fail, they then go to the symptomatic treatment, prescribing sometimes for acidity, sometimes for nausea, sometimes for flatulence, sometimes for constipation, or "the liver," or debility.

You should be careful in the examination of such cases, and should try to ascertain whether these symptoms may not depend upon inflammation of the stomach; for as long as the patient is in this state, the less you have recourse to symptomatic or specific treatment, the better. It is hard to mention one single medicine which in this state will not prove stimulant; and if the stomach be unfit for stimulants, it must be unfit for the generality of medicines. There are numbers of cases of persons laboring under chronic gastritis, which have been cured by strict regulation of diet, and by avoiding every article of food requiring strong digestive powers. We find that articles of diet vary very much in this respect; some are digested with ease, some with pain. We might express this otherwise, by saying, that some require very little excitement of the stomach, and others very great vascular excitement. Patients in this irritable state of stomach, can scarcely bear any kind of ingesta; and when you consider the great vascularity, thickening of the mucous membrane, and tendency to organic disease, you will be induced to think that every thing entering the stomach should be of the mildest kind, and not requiring any powerful determination of blood to that organ.

If you continually prescribe for symptoms, neglecting or overlooking the real nature of the disease, giving arsenic to excite the system, and iron to remove anæmia, and bitter tonics to improve the appetite, and alkaline remedies for acidity, and carminatives to expel flatus, you will do no good; you may chance to give relief to-day, and find your patient worse to-morrow; and at last he will die, and you may be disgraced. On opening the stomach after death, you are astonished to find extensive ulceration, or perhaps cancerous disease. Very often in such cases, practitioners say that it is cancerous disease, and that no good can be done. But the thing is, to be able to know, when you are called to a case, whether it is a case of mere nervous dyspepsia, or chronic inflammation of the stomach. Some of the best pathologists think that most of the cancerous affections of the stomach, are in the beginning only chronic inflammations of that organ.

I believe we have not yet in this country adopted the plan of moderate application of leeches to the epigastrium, in cases of chronic gastritis. I have seen in many cases great benefit result from the repeated application of a small number of leeches to the epigastrium, at intervals of two or three days. Here is a point which you will find very useful in practice. You will meet with cases which have lasted for a long time; cases where there is strong evidence of organic disease, and which have resisted the ordinary dyspeptic treatment. You will be called frequently to treat these three different cases,—where the disease has been of long duration, where there is distinct evidence of organic disease, and where the disease has resisted the ordinary dyspeptic treatment. Here is a case of a patient laboring under what is called indigestion, and which has resisted the stimulant, and tonic, and purgative treatment. Here is one fact. In the next place, the disease is chronic, and the probability is, that there is inflammation, and consequently that there is chronic gastritis. Now if in such a case you omit all medicine by the mouth, apply leeches to the epigastrium, keep the bowels open by injections, and regulate the diet, you will often do a vast deal of good. I have seen, under this treatment, the tongue clean, the pain and tenderness of the epigastrium subside, the acidity, thirst, nausea, and flatulence removed, the power of digestion restored, and all the symptoms for which alkalies, and acids, and tonics, and purgatives, were prescribed, vanish under treatment calculated to remove chronic inflammation of the stomach.

What is next in importance to regulated regimen and local bleeding?—A careful attention to the bowels, which in chronic gastritis are generally constipated, and this has a tendency to keep up disease in the upper part of the digestive tube. Is this to be obviated by introducing purgative medicine into the stomach?—No. If you introduce strong purgative medicine by the mouth, you will do a great deal of mischief. You must open the bowels by enemata, or if you give medicine by the mouth, by the mildest laxatives in a state of great dilution. A little castor oil, given every third or fourth day, or a little rhubarb with some of the neutral salts, will answer in most cases. The diet too, can be managed so as to have a gently laxative effect. The use of injections is, however, what I principally rely on. I have seen many cases of gastritis cured by the total omission of all medicine by the mouth, by giving up every article of food which disagreed with the stomach, and by the use of warm water enemata. I have seen this treatment relieve and cure persons whose sufferings had lasted for years previous to its employment, and who had been considered by eminent practitioners to labor under organic disease of an incurable nature. It is important that you should bear this in mind. The old purgative and mercurial treatment of gastritis, I am happy to say, is rapidly declining; and British practitioners are now convinced, that they cannot cure every form of dyspepsia by the old mode of treatment. I do not deny that many diseases of the digestive tube may be benefitted by the mild use of mercury and laxatives, but I think I have every reasonable and scientific practitioner with me, in condemning the unscientific routine practice, which was followed by those who took the writings of Abernethy and Hamilton for their guide. I do not say, that where cases of gastric inflammation, treated after the plan of Mr. Abernethy, have proved fatal, the medicines have destroyed life; I merely assert that the patients died of inflammation, over which these medicines had no control; and the error lay in mistaking and overlooking the actual disease, as much as in its maltreatment. You will find some practitioners, (they are becoming fewer in number every day,) who seem to have but two ideas, the one a purgative, the other a pot full of fæces; but the connecting link,—the gastro-enteric mucous membrane,—that vast expansion, so complicated, so delicate, so important, seems to be totally forgotten. But practitioners are now beginning to see that purgatives are not to be employed empirically; that they should be administered in many cases with great caution, and with a due attention to the actual condition of the alimentary canal, and that they have been a source of great abuse in the medical practice of these countries.

Next to leeching and the proper regulation of the bowels, is the employment of gentle and long continued counter irritation over the stomach. This may be effected by the repeated application of small blisters, or by the use of tartar emetic ointment. I have been in the habit of impressing upon the class, that the tartar emetic ointment used in these countries, is too strong, the consequence of which is an eruption of large pustules, which are excessively painful, and often accompanied with such disturbance of the constipation as amounts to symptomatic fever. In fact tartar emetic ointment of the ordinary strength, produces so much irritation that few patients will submit to it long.—The form which I recommend you to employ is the following:—Take seven drachms of prepared lard, and instead of a drachm of tartar emetic, which is the usual quantity, take half a drachm, directing in your prescription, (this is a point of importance,) that it be reduced to an impalpable powder; and you may add to it, what will increase its action, one drachm of mercurial ointment. This produces a crop of small pustules, which give but little pain and are easily borne; and the counter irritation may be kept up in this way for a considerable time, by stopping for a few days, until the eruption fades away, and then renewing the friction. I have often seen the utility of this remedy exemplified in cases of chronic gastritis, where the symptoms of gastric irritation, which had subsided under the employment of friction with tartar emetic ointment, returned when it was left off, and again vanished when it was resumed. The case of the celebrated anatomist, Beclard, furnishes a very remarkable proof of the value of a well regulated diet, and repeated counter irritation, in the treatment of this disease. While he was engaged in the ardent prosecution of his professional studies, he got an affection of the stomach, which he considered to be a chronic gastritis, and immediately put himself under a strict regimen, using at the same time repeated counter irritation.—He kept up the counter irritant plan for a considerable length of time, for he found, that when he discontinued it, the gastric symptoms had a tendency to return. In this way he got completely rid of the disease. Several years afterwards he died of an attack of erysipelas; and on opening his stomach, the cicatrix of an old ulcer was discovered in the vicinity of the pylorus, which was exactly the spot to which he had referred his pain during the continuance of the gastric affection.

Gentlemen, there is perhaps no science in which the motto "*medio tutissimus ibis*," is of more extensive application than in medicine.—Some physicians on the continent, particularly the disciples of Broussais, having repeatedly witnessed the advantages of strict regimen and local depletion, in chronic gastritis, have pushed this practice too far. They seemed to forget that the system requires support and nutrition, which can be effected only through the agency of the stomach; they saw the evils which result from the use of stimulating food, in cases of chronic gastritis; and looking to these alone, they ran into the opposite extreme; the consequence of which was, that they kept their patients so long upon low diet, that they actually produced the very symptoms which they wished to remove. The patients became dyspeptic from real debility of the stomach and the whole frame. You remember a general law of pathology, to which I have alluded on a former occasion, and which I shall again mention, as it illustrates this point; namely, that opposite states of the economy, may be accompanied by the same symptoms. Thus we observe, that palpitation may depend upon two different causes—on a sthenic or asthenic condition—on the presence of too much or too little blood in the heart. Now it frequently happened, that patients laboring under chronic gastritis, and who had been treated for a long time after the strict plan adopted by the Broussaists, finding themselves not at all improved, went to other physicians, who had different views, and were rapidly cured, by being put upon a full nutritious diet. In this way numerous cases, which water diet and depletion had only aggravated, were relieved, and the consequence was, that a mass of facts was brought forward and published, not long since, by a French author, against the antiphlogistic treatment of dyspepsia and chronic gastritis. It must be stated, however, that the cases which he published were chiefly those in which the depleting system had been carried to excess; and that they cannot therefore, be received as proofs of the value of a stimulating diet, in the treatment of chronic inflammation of the stomach. Bear this in mind; the sooner you can put your patient on a nutritious diet, the better will it be for him. It would be absurd to keep a patient for many months, as the Broussaists have done, on slops and gum water. It will be necessary for you to feel your way, and improve the diet gradually. Commence by giving a small quantity of mild nutritious food; if your patient bears it well, you can go on; if the gastric symptoms return, you can easily stop. If a small portion of the milder species of food rests quietly on the stomach, you may increase it the next day or the day after, and thus you proceed to more solid and nutritious aliment, until the tone of your patient's stomach regains the standard of health. Never lose sight of this fact, that you may have a case of dyspepsia, depending on a chronic gastritis, in which, though you remove the *inflammation*, by a strict antiphlogistic treatment, you may not by this remove the *dyspepsia;* and if you continue to leech, and blister, and starve your patient, *after the inflammatory state be removed*, you will do great injury. Such a patient, falling into the hands of another practitioner, who treated him on a different system, might be relieved, and his case quoted against you and your treatment, though this, at the commencement, was judicious and proper.

With respect to internal remedies, the school of Broussais think that there is nothing required but cold water and gum. This is going too far. In a former lecture, I have drawn your attention to the fact, that in the treatment of acute inflammation, there is a point where antiphlogistics should cease, and where tonics and stimulants are the most efficient means of cure. Of this fact, the disciples of Broussais appear to be ignorant, and they consequently declared against every remedy for chronic gastritis, except leeches and cold water. Now is this right? I think not. We find that, in all cases of gastric inflammation, a change in medication seems to be useful at some period of the disease; that is, a change from antiphlogistics to tonics and stimulants; and I believe that in cases of chronic gastritis, these remedies may be used with very great advantage, having of course premised depletion and counter irritants. I believe too, that most of the remedies which we see every day unsuccessfully employed, would have acted beneficially, if the preparatory treatment which I have mentioned had been adopted. Among the best remedies of this kind, is the oxide of bismuth; I have seen more benefit from the use of this than of any other medicine, after the treatment already alluded to.—Generally speaking, the list of internal remedies for chronic gastritis, is very small; but after the use of antiphlogistics, you may prescribe the vegetable tonics and oxide of bismuth with advantage.

The most decidedly valuable remedy, however, in the after stage of a chronic gastritis, is the acetate of morphia, which I am convinced has a very powerful effect in allaying chronic irritation of the stomach. Dr. Bardsley, of Manchester, in one of his published works, entitled "Hospital Facts and Observations," adduces many cases of gastric irritation which were completely relieved by the use of this remedy, and I am perfectly satisfied of the truth of his statements. It may be said, that Dr. Bardsley's cases were only instances of dyspepsia. But as his cases were extremely numerous, some of them of long standing, and the symptoms very severe, the great probability is, that some of them at least must have been cases of chronic gastritis. I know very few books, the perusal of which I would more strongly recommend to you, than Dr. Bardsley's accurate and instructive work. The great besetting sin of Medical writers is, that their statements of successful practice are grounded on a very limited number of cases; or that, in publishing the result of their practical investigations, they only give their successful cases, and leave out those in which the treatment recommended has been found inefficacious. Yet this is a circumstance which should never be neglected. If a man declares that he has discovered a cure for gastritis, or dyspepsia, and brings forward one hundred cases in which the remedy has done good, the statement is still unsatisfactory and insufficient, because there may be one thousand cases in which it has totally failed. Unless he comes forward and gives both his successful and unsuccessful cases, of what value are his statements? Dr. Bardsley, with the candor and good sense which always characterize the philosophic inquirer, gives the result of *all* his cases, forms them into tables, and then leaves his readers to judge for themselves. From an inspection of these tables, you will be convinced of the efficacy of acetate of morphia, in the treatment of chronic gastritis. I have been in the habit of using it, with the most gratifying results, after leeching, regulating the diet, and paying proper attention to the state of the bowels. There are some forms of the disease in which it is more useful than others. The particular form in which it proves most serviceable, is where there is a copious secretion of acid from the stomach, (that form in which all kinds of alkalies have been exhibited,) where severe pain and constant acidity are the prominent symptoms. Here I have seen the acetate of morphia act exceedingly well. You may begin with one-twelfth of a grain, made into a pill with crumb of bread, or conserve of roses, twice a day; the next day you may order it to be taken three times, and you may go on in this way until you make the patient take from half a grain to a grain and a half in the twenty-four hours.

I shall here mention the circumstances of a case, which I do not mean to bring forward as an instance of cure, but as an illustration of the extraordinary power which acetate of morphia possesses in relieving gastric irritation. A gentleman of strong mind, and highly cultivated intellectual powers, which he kept in constant exercise, got a severe chronic gastritis; his appetite completely declined; he had frequent vomiting of sour matter; fœtid eructations; and such violent pain in the stomach, that he used, when the attack came on, to throw himself on the ground and roll about in a state of indescribable agony. He applied to various practitioners, had several consultations on his case, and the opinion of the most eminent medical men was, that he had incurable cancerous disease of the stomach. These symptoms continued for several years, but for the last two or three years they were quite intolerable. He had repeated cold sweats, vomited every thing he took, even cold water, was reduced to a skeleton, and led a life of complete torture. Under such circumstances, he tried for the first time, by my advice, the acetate of morphia. He tried it first in doses of one-tenth of a grain, three times a day, and experienced the most unexpected relief. On the third day, all his bad symptoms were gone. He had no pain, no vomiting, no sweats; his spirits were raised to the highest state of exhilaration, and he thought himself perfectly cured. He went out in the greatest joy, visited all his friends, and told them that he had at last got rid of his tormenting malady. In the evening he joined a supper party, indulged pretty freely, and next morning had a violent hæmatemesis, to which he had been for some time subject.—All his old symptoms again made their appearance. He again had recourse to the acetate of morphia, and again immediately experienced relief; but the vomiting of blood again returned, so that he discontinued the remedy. This gentleman is now in the enjoyment of good health. He regulated his diet, left off all medicine by the mouth, used warm water injections, and thus recovered from his supposed cancer.

I do not bring this case forward as an instance of the curative effect of acetate of morphia, but as an instance of its powerful effect in allaying gastric irritation. I could adduce other cases, in proof of its value in the treatment of the after stage of chronic gastritis, and particularly of that form in which pain and acidity are the prominent symptoms; but I perceive my time has nearly expired. At my next lecture, I shall give some other particulars connected with this subject, and then proceed to the consideration of diseases of the small intestine.

In speaking of the employment of counter irritation, in cases of chronic gastritis, I forgot to mention the use of friction with croton oil, which has been found beneficial in many cases of chronic inflammation. It has been extensively used by many practitioners, in the treatment of chronic affections of the joints, and in various forms of pulmonary disease; and I have employed it myself in some cases of chronic gastritis, with benefit. I cannot say that the cases in which I have used it, presented all the symptoms of chronic gastritis, but they were certainly cases of chronic gastrodynia, with severe local pain, nausea, and loss of appetite. It is an excellent counter irritant, and gives very little pain. The mode in which I employ it is this,—take a few drops of croton oil, five or six for instance, drop them on the epigastrium, and rub them in with a piece of lint or bladder interposed between your finger and the skin, and the next day you have an eruption of small papulæ, which you can increase at will. There is one interesting circumstance connected with the use of croton oil frictions, which you should be made acquainted with. The liability to produce counter irritation, seems to depend upon the absorption or non absorption of the croton oil; if it be absorbed it will purge, but if it be not, it will produce counter irritation. In cases of this kind, therefore, where it produces the necessary degree of irritation in the skin, the chances are, that it will not act disagreeably, by bringing on catharsis. I have only seen one case where there were both the eruption and catharsis. This was a gentleman who had lately suffered from dysentery in warm climates.

I may also mention, that in convalescence from an attack of chronic gastritis, you must pay great attention to diet, for a long time, because there is no affection of any organ in the body, in which an error in diet so rapidly induces a return of the original symptoms, as in disease of the stomach, while each return of the disease renders the attack more dangerous and unmanageable, until at last disorganization takes place.—*Ibid., Feb.* 1*st and* 8*th,* 1834.—*Am. Journ. Nat. Sci.*

7. *Hydrocele cured by Tincture of Iodine.*—M. P. Record, Surgeon to the *Hopital des Vénériens,* has employed the tincture of iodine with success, in five cases of hydrocele independent of any syphilitic cause. The tincture of iodine is diluted with distilled water, and applied to the tumors by aid of compresses imbibed with it, and in which the scrotum is enveloped. M. Record employs the tincture in four different degrees of concentration:—1st. Tinct. iod ʒj.; Aq. distil. ℥iij.; 2d. Tinct. iod. ʒij.; Aq. distill. ℥iij.; 3d. Tinct. iod. ʒiij.; Aq. distill. ℥iij.; 4th Tinct. iod. ʒvj.; Aq. distill. ℥iij. In subjects whose skin is very delicate, and epidermis thin, the last formula is employed. When there is less sensibility, and some hardness of the tissues, the other formulæ are employed pro re nata. For the medicine to produce its beneficial effects, the patients must experience a rather vivid but supportable sensation of heat; and without there being burning or vesication, the skin of the scrotum must become brown, or pass into brownish red, the epidermis becoming like parchment, and forming scales that are detached leaving beneath them a sort of thick transpiration, still without vesication. So long as these results are not obtained the dose of the tincture of iodine must be increased, the quantity of distilled water remaining the same; but when these effects have resulted, the tincture of the same degree of concentration must be continued by steeping compresses in it, and renewing them twice a day. If pain supervene, the application is suspended for a few days, and its use resumed until the disappearance of the tumor.—*Journal des Connaisances Med. Chirurg. Jan.* 1833.—*Am. Journ. Nat. Sci.*

8. *Resection of Bones in Ununited Fractures.*—Surgeons have long entertained an opinion with regard to the resection of bones in cases of ununited fractures, which M. Dupuytren considers as very injurious. They imagined it was absolutely necessary to cut out both fragments of the bone, before a solid callus could be laid down; hence they avoided this operation in a great number of cases when this resection was extremely difficult if not impossible: thus, for example, they rarely had recourse to it in fractures of the femur, where the fragments frequently ride upon one another; the upper portion is the only one which can generally be got at with facility, while the inferior fragment, carried backwards and inwards, is too deep seated to be exposed by an incision in this direction, while the presence of important vessels prevents any operation on the inner side of the limb. M. Dupuytren is inclined to hold this opinion as altogether wrong, and maintains that the resection of one fragment is sufficient to obtain the consolidation of both; at least two cases in which he employed this practice turned out successfully.—*Lecons Orales.—Am. Jour. Med. Sciences*.

9. *Treatment of Fractured Limbs, by Inclosing them in Plaster Moulds, &c.*—The eminent German surgeon Diefenbach is in the habit of treating many cases of fracture, especially of the bones of the leg, by enveloping the limb, or only three-fourths of its circumference, with plaster of Paris, after it has been ascertained that the fractured extremities lie in normal apposition with each other. It is of advantage to leave the anterior part of the limb exposed, as we have thereby an opportunity of watching the progress of the cure, and of applying any local remedies which may be necessary, especially when the case is one of compound fracture.

The principle of Dieffenbach's plan, that of an unmoveable apparatus round the broken limb, has been adopted by many surgeons both of ancient and of modern times. It is to that glory of French military surgery, M. Larrey, that we are chiefly indebted for its establishment in present practice. In the Russian campaign, the method was most extensively adopted, and many a limb which had been fractured by gun-shot injuries, and had been thus treated, was found, on the return of the poor fellows to France, to have knit together most admirably, although the dressings had never once been removed, from the period of the accident. Larrey's apparatus has also the great advantage of being composed of materials the most simple, cheap, and almost always at command; for all that is required is a quantity of linen, straw, eggs, camphorated spirits, and of cold lead lotion. The mode of its adjustment is little more complicated than the application of a common bandage; and when once secured, the member is comparatively safe; and the patient, instead of being condemned to repose for a lengthened period, may be permitted to rise on the third or fourth day, and move about on crutches. Whatever be the limb which has been fractured, there is little risk of the apparatus being disturbed, even by a long journey; and if the jolting of the carriage be painful or improper, there is nothing to prevent the simultaneous employwent of suspension, as proposed by M. Sauter. But often this is not necessary; for in from twenty-four to thirty-six hours, after the apparatus has been applied, it forms a compact, coherent, and accurately fitting mould or case round the whole of the limb, so that no one part of it can be moved without the rest of the limb following the motion; thus all danger of lateral displacement is securely guarded against. It may be supposed that it is less efficacious in counteracting the shortening of the broken limb than some methods of permanent extension; this may possibly be true, but when it is stated that, out of eight cases of fractures of the femur, six were cured, without any shortening of the extremity, the argument cannot form any very potent objection to the practice; and indeed the very fact of all the muscles of the limb being equally and strongly compressed, (for the apparatus should be made to incase the whole of its length,) subdues in a great measure their tendency to contraction, and therefore obviates the tendency of the bones to ride over each other.

But if the immoveable apparatus which we have so strongly praised, and which our readers will find described at length in the third volume of Baron Larrey's Clinique Chirurgicale, and also in an admirable thesis published lately by his son, possessed no other advantages besides the one of permitting the patient to move about safely during the treatment of his fracture, we should deem this sufficient to recommend it to the notice of surgeons; perhaps a case in point will be received as the most satisfactory evidence of the benefits accruing from its adoption.

A man of healthy constitution was admitted into a hospital for a complete fracture of the bones of the leg, about two inches above the malleoli. During six weeks he was treated by the common method, but at the end of that time it was found that union had not taken place. The foot being considerably everted, M. Berard first brought it into its right position, and then retained it there by means of Dupuytren's splints for another month and a half; and although there had been no displacement all this time, there was still a complete failure of any bony union. The immoveable apparatus was now applied; and when this had become sufficiently firm and compact, the man was allowed to get out of bed and walk about the ward on his crutches, without ever putting the broken limb to the ground. After the expiration of six weeks a perfect cure was obtained.—*Med. Chir. Rev. from Archives Générales.—Am. Jour. Med. Sciences.*

10. *On Bony Union of Fractures of the Neck of the Thigh Bone.* By Sir Astley Cooper.—I find in a report of Le Baron Dupuytren's lecture, that he attributes to me the opinion, that fractures of the neck of the thigh bone, within the capsular ligament, not only "never unite, but that it is impossible they should unite by bone."

It is quite true that, as a *general principle,* I believe that those fractures unite by ligament, and not by bone, as do those of the patella and olecranon. But I deny that I have ever stated the impossibility of their ossific union; on the contrary, I have given the reason why they occasionally may unite by bone.

The following are my words—"*To deny the possibility of their union, and to maintain that no exception to this general rule may take place, would be presumptuous.*"

I then proceed to state that the cause of the deficient union is to be found in the reflected ligament of the cervix femoris being torn through, and that thus a defective nutrition of the head of the bone is produced. But I held, that if the reflected ligament remained whole, or was but slightly torn when the bone was broken, it would unite by means of its cancellated structure; but that in such a case the limb would not be shortened in the usual manner, nor would the case have the common characteristics of this accident.

In proof of the truth of these observations, I shall give the following quotation from Mr. Swan's work on the Diseases of the Nerves, page 304.

"Case.—Mrs. Powell, aged above eighty years, fell down in the afternoon of the 14th of November, 1824. I saw her soon after, and found her complaining very much of pain in the left hip. The limb could be moved in every direction, but this motion produced excessive pain. She lay on her back, with the limb extended; and nothing *was ever done*, except the application of fomentations for the first few days. I believed there was a fracture of the neck of the thigh bone, although the limb remained quite as long as the other, and I neither perceived a crepitus, nor any altered appearance in its position, except a slight inclination of the toes outwards. She had more constitutional irritation than I ever observed from a similar accident. She suffered much pain in her hip, and was in consequence obliged to take an opiate; but she got very little rest. She generally had much thirst. There was the utmost difficulty in keeping her bowels open; and she had great pain and difficulty in making water. She had no appetite for common food, and for three weeks appeared so weak, that she was under the necessity of taking wine and brandy. For some time, all her urine and stools were passed in bed, but not involuntarily, and only because she could not be persuaded to use proper means; in consequence, her back became very sore. Latterly she complained of pain in the abdomen, which was very tender on pressure, and made even the weight of the bed clothes inconvenient. Her tongue became very dry and brown; and in the last twenty-four hours she was insensible. She died on the morning of the 19th of December, about five.

"*Examination.* This took place at seven in the evening.

"There was some echymosis amongst the muscles about the injured part, and in the cellular membrane, about the sciatic and anterior crural nerves. The greatest part of the fracture of the neck of the thigh bone, which was entirely within the capsular ligament, was firmly united.

"A section was made through the fractured part, and a faint white line was perceived in one portion of the union, but the rest appeared to be entirely bone." Mr. Swan goes on to say, "This beautifully shows the principle which Sir Astley Cooper has advocated—viz.—that when the reflected ligament remains whole, and the bones are not drawn asunder, the nourishment to the head of the bone continues, and union will be produced even in the short space of five weeks, by only placing the knee over a pillow, and in other respects leaving the case to nature."

Of the contrary state of the fracture—viz. that in which the reflected ligament is torn—I may give the case sent to me by Mr. Robertson, of Sheerness, in which the patient's limb was extended for six months without any ossific union.

On the 25th of June, 1822, William Durain, aged 62, a tall, athletic convict, of a sanguine temperament, fell with a very inconsiderable violence, across a piece of timber in the dock yard, his left hip coming in contact with the wood. On rising he felt an acute pain in the region of the acetabulum, but no other inconvenience, for he walked on board to exhibit himself to the surgery man. From finding him ranked up with the sick of the hulk, on my morning visit of the 26th, from his walking on board, and from his own account of the accident, I did not suspect any serious injury to the joint, and treated the case as one of concussion. On the 29th, however, he complained of a very sudden and very agonizing accession of pain, which induced me to subject him to a more critical examination. No evident alteration in the size of either hip could be discerned, but a shortening of the limb was conspicuous, which was rendered more evident by making him stand upon the sound limb: extension removed this difference; but, on being freed from restraint, it again assumed its morbid shape; the knee and foot were everted, and rotation greatly increased his pain.

I removed him to the hospital, as a case of fracture within the capsule; but a continued attention for a period of six months to position, (chiefly with a view of restraining the motion of the pelvis, and of securing the limb,) made no other alteration in the symptoms than a gradual diminution of pain: a pair of crutches were given him, he was placed on the invalid list, and remained till the 26th of September, when he died from general dropsy. On dissection, the injury proved a transverse fracture of the head of the femur, within the capsular ligament; no species of union had taken place. The upper portion of the fractured bone was retained in situ, by the sound ligament, tolerably smooth upon its surface, but without any ossific doposit.

The lower portion was very irregular, with several detached pieces of bone adhering to the insertion of the capsular ligament. Between the acetabulum and the portion of bone retained in situ by the ligament, were several small, oval shaped, loose cartilaginous substances, apparently fragments of bone; the capsular ligament partially lacerated, in a line above the trochanter major, and greatly thickened in its insertions.

I should not have given you this trouble, nor should I have taken it myself, but for the respect I bear my friend the Baron Dupuytren; for, although I have already submitted myself to be misrepresented by many individuals, yet I should be sorry to be misunderstood by so excellent a surgeon, and so valuablo a friend, as le Baron Dupuytren. —*Medical Gazette, April 25th,* 1834.—*American Journal of the Medical Sciences.*

11. *Lithotomy.* [Extract of a letter from a physician in Paris, to the Editor.] I often attend CIVIALE's operations for stone, at the Hospital Necker. There seems to be no difficulty whatever in introducing the straight instrument into the bladder, and he sometimes succeeds in grasping the stone instantly; but very often he fails, and is compelled to postpone the operation on account of the pain experienced by the patient. I have seen his patients suffer more, apparently, from an unsuccessful attempt to break or drill the stone, than patients generally do from the operation with the knife. Agreeably to a report, made by Larrey and Boyer to the Academy of Sciences here, on the account rendered by Civiale, of his operations in the Hospital Necker, more than one third of his patients die, besides those that remain uncured. This operation, as practised here at present, is not attended with that success that is generally supposed in the United States.—There are a great number engaged in the operation, and each one has a different mode. From a report made to the Academy of Sciences by Baron Heurteloup, now operating in London, of his new instrument and its use, it seems that he has great success. M. Amussat has improved upon Heurteloup's instrument, and by his polite invitation, I have seen him operate with it frequently. This is the most simple, and I presume the best at present. It is composed of two pieces, and when prepared to enter the bladder, resembles a common sound. It is opened after entering the bladder; the stone is caught, and broken by percussion applied by means of a small hammer.

I have seen Amussat's patients suffer much from extracting the fragments of stone that lodge in the urethra after being broken to pieces.

I have taken some pains to collect all the information in Paris relative to the different modes of operating for stone. I have seen all the instruments that have been invented—have seen them tried often upon the dead subject, and the living, by their inventors, and those most skilled in their use, and I am now fully convinced, that the *lateral* operation is far preferable to any other mode practised at present. Notwithstanding the horrid success attending lithotomy, as performed by Dupuytren, in the Hotel Dieu, it is not so painful to witness or think of, as the operation of Civiale at Necker. Dupuytren loses one in four or five.

It is not only in the operation for stone that we see such mortality in Paris, but in all other capital operations. I saw Dupuytren amputate the thigh of a healthy looking girl, for what he called rheumatism of the knee joint. When done the operation, his hands were full of ligatures. The patient was sent off without any dressing to the stump. In two weeks the *cadaver* of the girl was brought in for Dupuytren to explain the cause of her death. The os femoris was bare for some inches.—*Transylvania Journal of Medicine.*

12. *The New Alkaloids.*—The numbers of the *Journal de Pharmacie* for February and March, contain some interesting particulars on several of the new alkaloids.

*Sarsaparilline.*—This substance (says M. Thubeuf) is regarded as the active principle of sarsaparilla. Like the original root, it communicates to water the property of frothing when agitated, and gives it that sharp bitter taste which we find in the aqueous or alcoholic infusions of sarsaparilla. When examined by the microscope, it presents radiated crystals, the layers of which converge toward their extremities, and it has no action whatever upon turnsole paper. Sarsaparilline, when pure, is white, without odor, and, if it contain no water, is tasteless. It is little soluble in cold water, but the whole is taken up by boiling water, which throws it down again as it grows cold. It is soluble in alcohol, either cold or warm, and the substance may be crystallized in this manner by evaporation. It may be precipitated from its alcoholic solutions, by the addition of a little cold water. This alkaloid is insoluble in ether, even at the boiling point, but is very easily dissolved in a warm mixture of alcohol and ether in equal quantities.

*Atrophine.*—This substance (say MM. Geiger and Hesse) already noticed by Brande, has been obtained from the atropa belladonna. It has presented identical properties when obtained from the same part of the plant by different processes. It is white, and crystallizable into bright transparent prisms, which have a tendency to group together; at the ordinary temperature, water takes up only a hundredth part of the mass. The aqueous solution of atrophine renders turnsole paper blue, when reddened by an acid. It acts on the organ of vision in the same way as belladonna, and even a very weak solution is sufficient to dilate the pupil, which remains in that state for a considerable time. Chlorine has very little action upon it, and it seems to form sub-saline compounds with the acids. The aqueous infusion of atrophine forms an abundant white precipitate with the aqueous infusion of galls, and it forms an orange yellow precipitate with the hydrochlorate of gold.

*Hyosciamine.*—This alkaloid is most easily obtained from the seeds of the hyosciamus niger. It crystallizes into transparent colorless needles. Its taste is acrid and disagreeable, like that of tobacco, and it has a powerfully poisonous action. The smallest quantity placed on the eye, produces permanent dilatation of the pupil; in an anhydrous state it is not alkaline, but becomes so by the addition of a small quantity of water. The salts of hyosciamine are neutre, they are partially crystallizable, and are as poisonous as the plant itself.

*Daturine.*—This alkaloid has been extracted by the same chemist from the datura stramonium. It readily crystallizes into regular prisms, which are colorless and brilliant. It is inodorous. The taste is at first slightly bitter, and then becomes very acrid, like that of tobacco. Daturine is a highly poisonous substance; one-eighth of a grain was enough to poison a sparrow in three hours. When placed on the eye, it occasions a strong and permanent dilatation of the pupil.

*Solanine.*—This substance, already discovered by several French chemists in the solanum nigrum, solanum dulcamara, and the solanum mammosum, has been recently found in the potato, by M. Otto, of

Brunswick. He obtained the alkaloid in the following manner:—The potato buds were first treated in water, acidified by sulphuric acid; he then separated the sulphuric and phosphoric acids, with the extractive matter, by acetate of lead; the colorless liquor which remained was saturated with lime water, and the precipitate boiled in alcohol; and the product thus obtained was purified by frequent washing in alcohol. In order to determine its action on the animal economy, M. Otto tried the effects of this substance upon rabbits, and found that it should be ranged amongst the acrid narcotic poisons. One grain of solanine was sufficient to kill a rabbit in six hours; a second rabbit, which was much stronger than the former, was destroyed in nine hours after having taken three grains. It exercises a remarkable paralysing effect on the hinder extremities of the animal before occasioning death, and this paralysis may be produced in horned cattle by simply feeding them on the washings of potato tops. This substance is white and powdery, and turns blue turnsole paper which has been reddened by the action of an acid. The greater part of its salts assumes an aspect like gum upon desication.

*Colchicine.*—This alkaloid crystallizes into delicate needles; its flavor is very bitter; when placed in the nares it does not excite sneezing, while the smallest portion of veratrine occasions a violent fit.—Colchicine is a strong poison. One-tenth of a grain dissolved in alcohol was given to a cat six weeks old; the animal began at once to foam at the mouth; after the lapse of an hour she passed abundant alvine evacuations, and vomited; she soon became feeble, unable to walk steady, fell down on the ground, rolled about, and was agitated by convulsive motions; these symptoms increased in severity; the animal died in twelve hours. On examining the body after death, the intestinal canal was found to be violently inflamed, and blood was effused into its tissue through the whole extent. One-twentieth of a grain of veratrine was given to a younger cat, in order to judge of its effects by comparison; they were very energetic; the animal quickly became feeble, was seized with convulsions, fell on the ground, and died within ten minutes. After death nothing remarkable was discovered, except inflammation at the upper part of the œsophagus.—This portion of the canal was not inflamed in the cat poisoned by colchicine.

*Aconitine.*—M. Geiger has found this alkaloid in the leaves of the aconitum napellus; it does not seem susceptible of crystallization.—When perfectly pure, it is white, grained, and forms a colorless, transparent mass. Its taste is bitter, then acrid, but the acridity is not strong or permanent. In this respect it differs from the plant, whose taste often remains twelve hours, leaving the tongue quite benumbed. The acrid principle is united with the aconitine, from which it may be separated by repeatedly combining the alkaloid with acids. When this substance is completely deprived of the acrid principle, it is excesively poisonous; the one-fiftieth part of a grain, dissolved in alcohol, was sufficient to kill a sparrow in a few minutes, and one-tenth of a grain destroys a small bird with the rapidity of lightning. When placed on the eye it produced dilatation of the pupil, but the effect is of short duration.—*French Gazette.—Boston Medical and Surgical Journal.*

13. *Longevity of Medical Men.*—It is a common impression, and one which has received confirmation from examples which have been presented to us in this immediate vicinity, that physicians, as a body, attain to more than the average duration of human life—that the cares, the anxieties. and the vexations to which this profession are subjected, are in some measure compensated by an extension of their term of existence, beyond what is usually allotted to mankind. Here in this city, and before our own eyes, the instances of medical longevity have been sufficiently numerous to encourage this idea; and the single fact that the venerable Holyoke nearly attained the great age of 101 years, was of itself sufficient for the foundation of an hypothesis. It might indeed seem surprising that this should be the characteristic of a profession, the members of which are not as a body endowed with stronger physical powers than the avarage; a profession which dooms one to a most irregular vicissitude of toils and labor, and unprofitable rest; which wages an implacable warfare with all uniformity and regularity of habits; which interrupts, defers, and abridges the meals, breaks in upon the hours of sleep, and often leaves scarce time for the absolutely necessary attentions to the person; which makes it almost impossible to meet with proper measures the incipient stages of disease; which exposes to the inclemencies of the weather, both in season and out of season, by night and by day; which at once wearies the body with toil and the mind with anxiety; which in fine involves an alternation of excitement and depression, of delight and despondence, far beyond what belongs to the even tenor of any other occupation: that such a profession should be favorable to long life, would, if true, be not a little surprising. And in order to account for this apparent anomaly, various explanations have been devised, having more or less plausibility, though all falling much short of the object aimed at. It has, for instance, been attributed to the peculiarly philosophical habits of mind induced by the profession, the equanimity and calmness which its duties tended to produce and to foster; a view, which, however applicable to some, is far from being so to all, or even the greatest number. It has also been said that peculiar temperance was the secret of the longevity of physicians—a position which, for the honor of the profession, we should be abundantly willing to admit. Others have thought that the physician, by timely precaution and watching the first intimation of disease, was able to prevent its advancing to any serious extent in his own person; a kind of care which in fact he is less able to exercise than almost any one. And, finally, not a few have thought that the whole secret consisted in the Doctor having the prudence to beware of those doses with which he so liberally supplies his patients. Whatever influence, however, all or any of these circumstances may have in prolonging the life of physicians, it seems altogether unnecessary to resort to them, unless the simple fact that this profession enjoy a longer reprieve than others from the common doom of mankind, can be made apparent. Such is certainly not the result of inquiry among our medical brethren abroad. We have now before us a very able paper by Professor Caspar, of Berlin, published in the Berlin Medical Journal, which goes expressly to establish the contrary conclusion, and to show that while among divines 42 out of 100 reach the term of three score and ten, and among members of the legal profession 29 out of 100, the proportion of medical practitioners who attain that age is but 24 in 100, or less than one-fourth!

In order to furnish data on which to found just conclusions in regard to this matter, Professor Caspar has ascertained the period of decease of 624 individuals of the medical profession in Germany, and therefrom has constructed a table on the followiug principle. The table consists of four columns. The first contains a series of numbers,

| A. Age. | B. Deaths. | C. Living. | D. Probable Life. | A. Age. | B. Deaths. | C. Living. | D. Probable Life. |
|---|---|---|---|---|---|---|---|
| 23 | 2 | 624 | 35.4 | 58 | 10 | 317 | 11.0 |
| 24 | 1 | 622 | 34.4 | 59 | 17 | 307 | 10.6 |
| 25 | 4 | 621 | 35.4 | 60 | 12 | 290 | 10.3 |
| 26 | 3 | 617 | 33.0 | 61 | 15 | 278 | 9.7 |
| 27 | 7 | 614 | 32.0 | 62 | 14 | 263 | 9.0 |
| 28 | 5 | 607 | 31.4 | 63 | 19 | 249 | 8.8 |
| 29 | 5 | 602 | 30.1 | 64 | 20 | 230 | 8.5 |
| 30 | 5 | 597 | 29.8 | 65 | 11 | 210 | 8.0 |
| 31 | 11 | 592 | 28.6 | 66 | 18 | 199 | 7.5 |
| 32 | 8 | 581 | 28.0 | 67 | 6 | 181 | 7.2 |
| 33 | 11 | 573 | 27.6 | 68 | 16 | 175 | 6.5 |
| 34 | 11 | 562 | 26.8 | 69 | 9 | 159 | 6.0 |
| 35 | 8 | 551 | 26.0 | 70 | 17 | 150 | 5.5 |
| 36 | 7 | 543 | 25.5 | 71 | 11 | 133 | 5.4 |
| 37 | 8 | 536 | 24.7 | 72 | 15 | 122 | 5.0 |
| 38 | 14 | 528 | 24.0 | 73 | 14 | 107 | 5.0 |
| 39 | 8 | 514 | 23.5 | 74 | 13 | 93 | 4.7 |
| 40 | 9 | 506 | 22.7 | 75 | 10 | 80 | 4.6 |
| 41 | 11 | 497 | 22.0 | 76 | 9 | 70 | 4.4 |
| 42 | 6 | 486 | 21.3 | 77 | 8 | 61 | 3.9 |
| 43 | 8 | 480 | 20.3 | 78 | 10 | 53 | 3.3 |
| 44 | 8 | 472 | 19.7 | 79 | 4 | 43 | 3.0 |
| 45 | 11 | 464 | 19.0 | 80 | 11 | 39 | 3.0 |
| 46 | 4 | 453 | 18.2 | 81 | 6 | 28 | 4.0 |
| 47 | 14 | 449 | 17.3 | 82 | 3 | 22 | 4.0 |
| 48 | 11 | 435 | 16.7 | 83 | 3 | 19 | 4.0 |
| 49 | 12 | 424 | 16.0 | 84 | 2 | 16 | 4.0 |
| 50 | 13 | 412 | 15.4 | 85 | 3 | 14 | 4.0 |
| 51 | 8 | 399 | 15.0 | 86 | 2 | 11 | 3.5 |
| 52 | 11 | 391 | 14.2 | 87 | — | 9 | 2.6 |
| 53 | 10 | 380 | 13.5 | 88 | 2 | 9 | 2.0 |
| 54 | 18 | 370 | 12.8 | 89 | 4 | 7 | 1.5 |
| 55 | 14 | 352 | 12.6 | 90 | 1 | 3 | 0.5 |
| 56 | 13 | 338 | 12.4 | 91 | 2 | 0 | 0 |
| 57 | 8 | 325 | 11.9 | 92 | — | 0 | — |

commencing with 23, the earliest age at which any decease is reported to have taken place, and extending to 92, which period no one of the 624 was destined to survive. The second column contains the number of deaths which took place at each of these ages, the sum of which completes the number 624. The third represents the survivors after the successive subtractions of the deaths, and the number in it go on diminishing from 624 to zero. The fourth represents in numbers the probable duration of life at each age, as deducible from the facts furnished by the table itself. The mode in which the numbers in this column are ascertained, is perfectly simple. It is done merely by counting off the number of lines to the period at which the survivors at the age in question, are reduced to one-half. For instance, at 25 the substractions have already reduced the number to 621. One half of this number is 310 1-2. In looking along the table we observe that the nearest number above this, viz. 307, corresponds to the age of 59 years, that is, at the age of 59 years, the survivors at 25 are already reduced to one-half. Of course the age of 59 is that which the physician at 25 has an equal chance of attaining. In general terms, if $m$ be the number of persons who have attained the age $a$, and at the age $n$ the number is found to be reduced to $\frac{m}{2}$, $n$ will express the probability of vital duration, and $n—a$ the number of years on which the individuals $m$ may calculate. "Of course," says the Professor, "nothing can be predicated from the table respecting the probable duration of the life of any individual; but the general conclusions, as experience has proved, are nevertheless extremely certain."

We have already alluded to the calculation made by Professor C. respecting the comparative chances of life among physicians, jurists, and theologians. The latter table will show more particularly how the account stands between the first and last of these professions.

| Ages. | Deaths. | |
|---|---|---|
| | Physicians. | Divines. |
| 23 to 32 | 82 | 43 |
| 33 — 42 | 149 | 58 |
| 43 — 52 | 160 | 64 |
| 53 — 62 | 210 | 182 |
| 63 — 72 | 228 | 328 |
| 73 — 82 | 141 | 255 |
| 83 — 92 | 30 | 70 |
| | 1000 | 1000 |

*Effects of Iodine.*—By Dr. Graves, of Dublin.—"If it be true," says Dr. Graves, "that mercury may be made to affect the mouth more rapidly by means of giving sulphate of quinine to the patient during or after its use, as has been asserted by Doctor Harty of Dublin, there must be some remarkable difference between the effects of sulphate of quinine and iodine on the animal economy, a difference well worth investigating, inasmuch as these remedies are usually considered very similar in their mode of action, and are often ordered indifferently in the treatment of chronic diseases, such as scrofula. As Dr. Kluge observes, the modifying powers iodine exerts on the action of mercury, opens a new field of inquiry in the treatment of the venereal disease, and renders it an object of great interest to discover to what species or stages of syphillis one or both of these remedies is adapted.

Doctor Kluge's experiments are of particular interest, when viewed in combination with the great encomiums recently bestowed in France and in London on the efficacy of iodine and deutiodide of mercury in the cure of syphilis and various other diseases. We have seen that two remedies, usually classed together as tonics, differ most materially in their effects, sulphate of quinine and iodine. The former stops the paroxysm of ague and induces salivation; the latter does not stop ague, and checks mercurial ptyalism. Again, when during a course of iodine, the patient becomes subject to pain in the stomach; this, as Lugol has shown, is best remedied by sulphate of quinine!

What are the therapeutical relations of another tonic, arsenic and iodine?—The former certainly exerts a powerful influence in controlling ague, and I have seen it produce salivation. Would iodine be useful in guarding against or mitigating its occasional bad effects?—If experience answered in the affirmative, no greater boon could be conferred on the practitioner, who would then be enabled to use long-continued arsenical courses in lepra, psoriasis, and various other diseases of the skin, without fear of producing unpleasant or dangerous effects on the constitution."

In this brief passage there is much to give us pause. Dr. Graves has founded some curious conjectures on more curious statements. Our business is chiefly with the latter.

Dr. Harty, of Dublin, would seem to have asserted, that sulphate of quinine, given before or after the exhibition of mercury, accelerates the affection of the mouth by the latter. That this is opposed to common observation is merely an obvious truism. The experience of all surgeons and physicians has shown them that measures of depletion always augment the influence of mercury. Bleed a man and keep him low, and mark how soon salivation is effected. Reasoning would lead the reflecting practitioner to suppose that converse circumstances would produce the opposite effects. And facts appear to warrant the suggestions of experience and judgment. We suppose it will be granted that few situations afford more ample opportunities of watching the effects of mercury than the wards of a Lock Hospital. Observation in those wards does not confirm the ideas of Dr. Harty. If a patient is weak, he is ordered the sasaparilla or the quinine, in order that he may *bear* mercury, and not be rapidly salivated by it. Again and again have we given quinine with this view and with the same result. We join issue with Dr. Harty. We maintain that cæteris paribus, quinine tends to prevent and not to produce salivation.

Dr. Graves observes that iodine and quina are usually classed together as tonics. They may be *classed* together in the schools, but the brittle and unsubstantial junction is like that of Nebuchadnezzar's image, one part iron and the other clay. They may be rivetted—they never can amalgamate.

The best mode of testing the real action of a remedy, is surely to push it to the point of producing injurious effects. Apply this test to iodine and quina. The patient who takes the former grows pale, emaciated, nervous—has flutterings at the epigastrium—a tendency to syncope—a tremulous and frequent pulse. He rallies under stimulants. Is this the action of a tonic? The man who is under the influence of quina displays very contrary phenomena. The tongue is probably loaded—the face is flushed—the head may ache, and fulness in it is experienced—the pulse is full—the vascular system is excited and replete.

In these two series of effects we perceive no resemblance nor any analogy. The obvious deduction from their observation is that, cæteris paribus, iodine debilitates and quina strengthens.

Perhaps Dr. Harty or others may urge that though iodine, pushed to a certain extent, occasions the results we have described, smaller and more guarded doses possess less exceptionable tonic properties. They may probably refer to the instance of opium, which, in moderate quantities, is said to be a stimulant, and a powerful sedative in larger. We cannot allow them the benefit of this analogy, nor the use of this conjecture. We have seen iodine employed extensively, but we never yet saw it act as a tonic. We have indeed seen patients received into a hospital, every circumstance altered in their favor, and iodine given in small doses. Such patients have gained flesh and acquired strength, but we could not avoid entertaining the suspicion that the results were vicious, the experiment deceptive. Even in those instances, the most favorable we have witnessed, the mineral was seldom continued long, without the supervention of unpleasant symptoms. So soon as the beneficial influence of better or of regulated diet, of repose, and of appropriate attentions had worn off, the depressing action of the iodine was more and more apparent.

There is a fallacy attending the operation of remedies like iodine, mercury, &c. which deserves to be adverted to. If they tend to effect the removal of a disease which is wearing down the powers of the patient, they act pro tanto and pro tempore as tonics. This is not unfrequently the case with mercury, which, under ordinary circumstances, undoubtedly displays no tonic properties. Nothing is more common than to see a person affected with secondary, or even neglected primary, symptoms, acquire strength and embonpoint so soon as mercury is properly administered.

Our readers may gather from these remarks that we do not consider iodine a tonic in the fair and general acceptation of the term; nay, we deem the notion pregnant with danger, and lift our voice most earnestly against it. The inexperienced practitioner should know that in using iodine he wields a potent and a deadly weapon. If he employs it as he would a tonic, he will probably find occasion to lament his error.

There are two particular statements to which we shall allude before we close these imperfect observations. The first relates to the success derived from iodine in secondary symptoms. Dr. G. observes that great encomiums have recently been bestowed on it, in reference to these affections, by surgeons in London and in France. Of this we are aware, and had the pleasure of conversing with some who have advocated the remedy in public and in private. We have given it in several cases of node, and in some of cachectic ulceration of the skin and of the throat. We have also seen it frequently exhibited by others. Yet such has been our evil fortune, that we never *saw* it prove of service. It was lately prescribed for a patient with node, in the Lock Hospital. The man took five minims three times daily, and had a meat diet in addition. After the medicine had been continued for ten days or a fortnight, the patient became so debilitated and depressed, that the iodine was instantly desisted from, and brandy and amonia substituted for it. In many instances it has seemed to do neither good nor harm; but in those the dose was small, and the patients had beer and good diet, which might probably counteract any mischeivous effects. On the whole, we feel no confidence in iodine as a remedy in syphilitic symptoms.

The second statement relates to the administration of iodine in cases of mercurial salivation.

Now this is observed under three sets of circumstances. In one, the patient is in tolerable health, and salivation results from a very inconsiderable quantity of mercury. It is said to depend, in such a case, on peculiar individual idiosyncrasy, and possibly the notion is correct.

In a scond instance, salivation is the consequence of mercury, actively pushed for an acute inflammatory malady, as hepatitis or phrenitis.

In a third, salivation is induced by a moderate quantity of mercury, given as a course for syphilis, or for any other disease. In this case, the patient is almost always weak, or his constitutional powers are impaired, or he is kept too low, or his room is warm, or some analogous cause exists. We had under our charge a case which illustrates this position. A female who had drunk great quantities of gin was affected with tubercular eruption, after a primary sore. We put her on the pilula hydrargyri, five grains twice daily, and conjoined with it a diet of animal food. In a very few days she became salivated, and had some symptoms of the mercurial erythismus. We discontinued the mercury of course; but as soon as we thought she could again support it we resumed its exhibition, adding porter to the diet. Again and again the same set of symptoms occurred. It struck us that the stimulus to which the patient had been previously habituated might enable her to bear the debilitating action of the mercury. We directed her to take a glass or two of gin during the day, and immediately a change for the better was observed, and the mercury was taken without inconvenience.

Thus, then, we remark that salivation may result from a small amount of mercury and peculiar idiosyncrasy—from mercury rapidly thrown in for acute inflammation—and from ordinary doses in debilitated patients.

Supposing iodine to possess the power of checking salivation, we apprehend that it would be applicable only to cases of the first description. In the second class, salivation is probably salutary, and, at all events, its speedy arrest would be dangerous, if not destructive. In the third class, salivation is the consequence of weakness and exhaustion, and we need not repeat our reasons for believing that iodine is not the remedy for *those* conditions. Fresh air, good diet, beer, and stimulants, are infinitely more adapted to them.

If iodine has really the virtue ascribed to it, that virtue may be safely and judiciously exerted in instances of salivation dependent upon idiosyncrasy. The cautious reader may hesitate in admitting even this as proved. We can only refer to the assertions of Professor Helmenstreitt, and the corroboration they receive from Dr. Graves. We are irresistibly inclined to acknowledge a degree of doubt.—*Med. Chir. Review.—Boston Med. and Sur. Journ.*

15. *Statistics of Accouchments which have taken place at the Maternité, Paris, during four years.*—From June 1829 to June 1833, there were born 10,742 children. Of this number 10,262 presented the summit of the head; 300 the pelvic extremities; 59 some portion of the trunk; and 30 the face. Of the children born by the head, 9,867 were born at the full period, 30 being brought forth dead. 395 were born before the full term. Of this last number, 34 had not reached the seventh month, and were not, consequently, viable; 89 were born in a putrid state. There only remained, therefore, 278 born before term, which could have lived, and, of these 48 died (one in five or six.) Amongst the 391 cases of pelvic presentations, 238 were at term, and 153 before it. Of the former 7 were born dead, having been for some time dead *in utero*. Of the 231 which remained, 21 were born dead (one in eleven). Of the 153 born before term, 63 were dead at an early period, and 30 were born at a time when they were not viable. Of the 60 viable which remained, 10 were born dead, which gives a proportion of one to six.—*Jour. Des. Con. Med.—Boston Med. and Sur. Journ.*

16. *General Statistics of Births.*—The following account, taken from the *Journal of the Statistical Society of France*, gives a curious scale of the proportion of male and female births in Europe; the result is the more worthy of attention, as it has been drawn from a scale comprising more than 70 millions of births, in which the cases have been meted:—

For every 100 girls born, there are born in Russia 108,91 boys. In the provinces under Milan, 117,61. In France, 106,55. In Holland, 106,44. In Pomerania and Brandenburg, 106,27. In Sicily, 106,-

18. In Austria, 106,10. In Silicia, 106,05. In Prussia, 106,94. In Wurtemburg, 105,69. In the Duchy of Posen, 105,65. In Bohemia, 105,38. In Great Boitain, 104,75. In Sweden, 104,66. Giving a mean proportion of 116 boys for every 100 girls. The two extremes are formed by Russia and Sweden, and the same results have always been observed in the same country.—*Boston Med. and Sur. Journ.*

17. *Treatment of Acute Rheumaism.*—A writer in the Medical Magazine has discarded bleeding in acute rheumatism, and uses a solution of tartar emetic and tincture of digitalis in its stead; the former given every sixth hour in as great doses as the stomach will bear without vomiting, and the latter every sixth hour in doses of from twenty to thirty-five drops. Several bad cases he has found to yield to the continued action of these two articles.—*Boston Med. and Sur. Journ.*

